# Books and Authors

**Published:** 1907-04

**Authors:** 


**American Practice of Surgery.** A Complete System of the Science and Art of Surgery by representative surgeons of the United States and Canada. Edited by **Joseph D. Bryant,** M.D., and **Albert H. Buck,** M.D., New York. Complete in eight volumes. Vol. II. Imperial octavo, pp. 778, illustrated. New York: William Wood and Company. 1906. (Price, Cloth: $7.00).

Scarcely had subscribers and others finished reading the first volume of this great system of surgery,—certainly they had not digested it,—when the second volume appeared. This one, like its predecessor, is divided into five parts, which are numbered consecutively to those in the first book. This, therefore, begins with part six, which deals with diseases that belong in varying degrees to the domain of surgery, and which are observed in certain parts of the United States and its dependencies, and in Canada. They are leprosy, plague, glanders, anthrax, actinomycosis, mycetoma, rhinopharyngitis mutilans, and scurvy, with special reference to diagnosis and surgical treatment. The section is written by James Farquharson Leys, Surgeon U. S. Navy. It is a most interesting and instructive disquisition on these several topics, that are too little studied by most surgeons. The illustrations are rare exhibitions of typical conditions found in each disease.

The next section, or part seven, gives a general survey of tuberculosis and syphilis in their relations to surgical work. The chapter on tuberculosis is written by Virgil P. Gibney and that on syphilis by Edward L. Keyes, Jr. The fact that tuberculosis invades so many tissues of the body not pulmonary, makes it quite as much a surgical as a medical disease; indeed, at the present day surgery is even more often invoked for the cure of tuberculous lesions than medicine. Gibney sets forth these and many other points regarding the modern pathology and treatment of the surgical aspects of the disease in forceful English, and with scientific effect. While, therefore, tuberculosis often falls within the domain of the surgeon's knife, syphilis, on the other hand, is a disease that concerns the surgeon as to diagnosis more than regarding treatment. Dr. Keyes makes this appear clearly in his discussion of syphilis, as well as the further fact that in late bone syphilis, where the destruction of bone by syphilitic inflammation is great, surgical intervention is the only way.

The succeeding section, part eight, deals with surgical diseases of various widely distributed strictures of the body, and include abscess, ulcer and ulceration, gangrene and gangrenous diseases, surgery of diseases of the skin; surgical diseases of wounds of muscles, tendons, bursæ, and connective tissue; surgical diseases of wounds of nerves, and surgical diseases and wounds of lymph nodes and vessels. The authors of these several topics respectively are August F. Jonas, William McDowell Mastin, Alfred C. Wood, Douglass W. Montgomery, J. Clark Stewart, DeForest Willard, and Charles N. Dowd. Montgomery's article on surgical diseases of the skin, covering nearly a hundred pages, is one of the most complete and best illustrated monographs on this topic that has been published in this country.

Coming now to part nine, we find dissertations on surgical diseases caused by intense heat and intense cold, and by the electric current written by Benjamin T. Tilton and Paul Monroe Pilcher respectively, both articles being concise and brief, but adequately handling these important injuries and their resultant lesions.

Finally, in part ten, simple and complicated wounds, including gunshot wounds, are presented with an exactitude as well as simplicity of detail, both charming and instructive. Captain Carl R. Darnall, Assistant Surgeon United States Army, wrote the article on wounds of soft parts by cutting and piercing instruments, and Major William C. Borden, Surgeon United States Army, wrote that upon gunshot wounds. The latter monograph is the most comprehensive disquisition upon gunshot wounds ever published in a general treatise on surgery. The volume is replete with sound instruction, a creditable production from American Surgeons, and a praiseworthy addition to surgical literature. The editors deserve especial commendation for their labors in successfully launching this great enterprise.

---

**Progressive Medicine, Vol. IV, December, 1906.** A Quarterly Digest of Advances, Discoveries and Improvements in the Medical and Surgical Sciences. Edited by Hobart Amory Hare, M.D., Professor of Therapeutics and Materia Medica in the Jefferson Medical College of Philadelphia. Octavo, 349 pages, with 29 engravings. Per annum, in four cloth-bound volumes, $9.00; in paper binding, $6.00, carriage paid to any address. Lea Brothers & Co., Publishers, Philadelphia and New York.

This issue of the quarterly digest completes the series for 1906. It is found a fitting climax to the excellent work which preceded it. The contributors have finished their labors for the year with never failing energy and care. As a result the busy practitioner can cull in an hour what it has taken investigators and teachers the world over months, if not years, to acquire. Special stress has been laid on practical results and the best methods of treatment. J. Dutton Steele devotes one hundred and twenty pages to diseases of the digestive tract and allied organs. His presentation of the subject, his attention to the minutest details and his clear deductions must prove suggestive to every reader. His handling of the causes of lowered gastric secretion are particularly forceful. The role of radiography in the diagnosis of gastrointestinal disease is clearly defined, and the employment of the Schmidt trocar and the Schmidt method for injecting salt solutions directly into the peritoneal cavity open up great possibilities for the future.

William T. Belfield treats of genitourinary diseases from tuberculosis to the irrigation and drainage of the seminal duct and vesicle. Under the diagnosis of renal disease, Kapsammer's report of over twenty thousand autopsies in Austrian hospitals, during the last ten years, is quoted. In regard to the relation of gonorrhea to disease of the sexual organs in women, he says: "While it is doubtless wiser for physicians to exaggerate than to minimize the remote evils of gonorrhea in both sexes, yet there can be no doubt that the current tendency is to be sensational rather than accurate in dealing with this question." His summary of the bactericidal effects of silver compounds; the various operative procedures for the relief of prostatic hypertrophy, and the cure of hydrocele, makes most interesting reading.

John Rose Bradford, in dealing with diseases of the kidneys says: "Functional albuminuria is a condition of great importance to all practitioners, both from a point of view of diagnosis and of treatment. Certainly, it would seem at the present time that many of these cases are submitted to far more rigorous treatment, especially in diet, than is necessary. Paroxysmal hemoglobinuria, the retention of chlorides in nephritis, and more particularly the treatment of nephritis are carefully considered. This section concludes with an article on renal dropsy.

Joseph C. Bloodgood devotes one hundred pages to fractures, dislocations, amputations, and the surgery of the extremities. The healing of fractures, the problems presented by fractures at the lower end of the humerus, metacarpel fractures, and sprains of the anklejoint are all thoroughly discussed. So with osteomyelitis, Paget's disease, and bone tumors. Bier's treatment with hyperemia is detailed at some length, and this section is freely illustrated. His conclusions in regard to the treatment of tuberculosis of the bones and joints are: "I believe, if these patients come to treatment in the early stage, that open air combined with Bier's hyperemia and proper orthopedic apparatus will accomplish an ultimate cure in an increasing proportion of cases, and operations will be performed, relatively, less frequently." H. R. M. Landis contributes over sixty pages to the therapeutic referendum, every page bristling with therapeutic facts of practical importance. He says: "It has now been established beyond a reasonable doubt that in tetanus antitoxin we possess a certain means of preventing tetanus, and that this serum should be used after all wounds in which there is any possibility to tetanus developing." Many useful suggestions are made in regard to drugs. Cases of poisoning by so-called "headache powders" are cited and the possibly injurious effects of adrenalin if used continuously. Alcohol, ergot, iodine, mercury, nitre, opium, the salicylates, urotropin and many other drugs, are dealt with, mushroom poisoning is handled, and many important facts are logically developed.

**International Clinics.** A Quarterly of Illustrated Clinical Lectures and especially prepared articles on Treatment, Medicine, Surgery, Neurology, Pediatrics, Obstetrics, Gynecology, Orthopedics, Pathology, Dermatology, Ophthalmology, Otology, Rhinology, Laryngology, Hygiene and other topics of interest to students and practitioners. By leading members of the medical profession throughout the world. Edited by **A. O. J. Kelly**, A.M., M.D. Volume IV, Sixteenth series. 1906. Philadelphia and London: J. B. Lippincott Co. (Cloth, $2.00).

The contents of this volume of the clinics present a varied and interesting group of lectures and papers. They comprise, in treatment, electrotherapeutics, by John H. W. Rhein; prevention and treatment of chronic nephritis, by James M. French; etiology and treatment of chronic constipation, by J. Dutton Steele; treatment of obesity, by P. Grocco; how operative treatment of renal or urethral calculi causes colic, by Howard Lilienthal.

In the department of medicine the topics are, clinical observations on the care of tuberculous subjects by William Porter; pulmonary tuberculosis in the middle aged and the aged, by J. Edward Squire; recent additions to knowledge of the physiologic influence of lowered barometric pressure, by Henry Sewall; obscure renal hematuria, by Arthur R. Elliott; myxedematous infantilism and incomplete myxedema, by Roger S. Morris; syphilitic aoritis, by C. Dieulafoy; recent progress in disorders of the adrenals, by Leon Bernard.

In surgery the subjects dealt with are, principles of treatment of fractures of the lower extremity, by George G. Ross; suggestions in the treatment of hip-joint disease, by Russell A. Hibbs; tuberculous hip-joint disease,—osteomeyelitis and transplantation of the fibula,—and ankylosis of the jaw, by James H. Rinehart; vesical tumors, by David Wallace; treatment of hemorrhoids, by George P. Miller.

In obstetrics and gynecology we observe, first, placenta previa and its treatment, by Joseph B. De Lee; next, lacerations of the cervix of the uterus and their immediate repair, by W. A. Newman Dorland; and finally, management of chronic cystitis in the female, by Daniel H. Craig.

Laryngology is represented by the laryngological complications of pulmonary tuberculosis, by E. F. Trevelyan; and otology by the mastoid operation, by Charles W. Richardson.

The articles by De Lee and Dorland are admirably illustrated, besides being clear and strong in the text. De Lee has presented the best exposition of placenta previa we have seen and, without disparagement to the other articles, we regard it the most attractive one in the book. The volume gives more literature of value for the money than any other serial with which we are familiar.

**Diet in Health and Disease.** By **Julius Friedenwald,** M.D., Clinical Professor of Diseases of the Stomach in the College of Physicians and Surgeons, Baltimore; and **John Ruhrah,** M.D., Clinical Professor of Diseases of Children in the College of Physicians and Surgeons, Baltimore. Second Revised Edition. Octavo of 728 pages. Philadelphia and London: W. B. Saunders Company. 1906. (Cloth, $4.00 net; half morocco, $5.00 net).

As must be the case in new editions this book has been revised and enlarged, which may mean much or little according to the way the reader views it. The section on salts has been rewritten and a feature is made of Prochownick's diet in pregnancy when complicated by contracted pelvis. Interesting are the diets in vogue at the various water cures and of particular interest is Klemperer's work on oxaluria. Yet, as with the majority of works on diet, there is too much that is confusing as well as too much that is left to the imagination of the reader, so far as the tabular work is concerned. It is not a criticism of this particular work to say that there is too much "maybe" and too little "is." That is the fault of nearly all diet books. Except in comparatively rare instances where there is no escape, no definite statement is made as to what must be avoided. There appears to be a general uneasiness and uncertainty in the maze of chemical constituents of the various foods. Merely as an illustration, if one were working out a diet list for an oxaluric individual, he would find that gooseberries were all right so far as oxalic acid was concerned, but that the calcium table would show them to be fairly rich in lime, hence not especially desirable from that viewpoint. Yet, after all is said and done, after the tables have been well digested and the diet list prepared, along comes the statement, based on experiment, that diet has little or nothing to do with the formation of calcium oxalate crystals; that they were found in fairly large quantities in the urine of a fasting dog. Aside from these criticisms, which as previously mentioned are not confined to this book alone, but are very general, it is most complete in the research line and leaves little to be desired as a really commendable work of reference on diet.

---

**The Ear and Its Diseases.** A textbook for students and physicians. By **Seth Scott Bishop,** B.S., M.D., LL.D., Honorary President of the Faculty and Professor in the Post-Graduate School and Hospital of Chicago; Surgeon to the Post-Graduate Hospital and to the Illinois Hospital, etc. Illustrated with 27 Colored Lithographs and 200 Additional Illustrations. Royal Octavo, 440 pages. Philadelphia: F. A. Davis Company. (Price, $4.00 net).

The author of this treatise is already widely known to the profession through his work on diseases of the nose, throat, and ear. In that book the ear was treated in too limited a manner to make it a desirable guide. Besides, diseases of the ear have assumed an importance of late, not hitherto accorded them. This is because they are becoming better understood. The anatomy of the ear is being more thoroughly studied and, as a consequence its pathology has become revealed more completely. These conditions have led to a better line of treatment in many affections not heretofore believed to be amenable to therapeutics, medical or surgical.

Bishop has presented his subject systematically, devoting five chapters to the anatomy and one to the physiology of the ear. He then takes up the examination of patients, the uses of compressed air, and next reaches the consideration of the diseases and injuries of the ear. These he discusses with great clearness, in some instances with minute detail, and always with scientific reason and research.

The previous work of Professor Bishop on "Diseases of the Nose, Throat and Ear," met with an enthusiastic reception and a large sale. The present treatise is the outgrowth of the author's experience in preparing the former book, and of his further experience in the field covered by this one; and it is fair to presume that this later effort will meet with equal approbation. It is written for the student and general physician; it is presented in a concise, plain and copiously illustrated manner; and these qualities so happly blended should insure its success.

---

**Abdominal Operations.** By **B. G. A. Moynihan,** M.S. (London), F.R.C.S., Senior Assistant Surgeon at Leeds General Infirmary, England Second Revised Edition, greatly enlarged. Octavo of 815 pages, with 305 original illustrations. Philadalphia and London: W. B. Saunders Company, 1906. (Cloth, $7.00 net; half morocco, $8.00 net).

Mr. Moynihan is not only a hard worker as a surgeon but he is industrious as a writer, his literary productions being as clean-cut as his surgical operations and their results. Only a few months intervened between the issue of the first edition of his treatise on abdominal operations and the demand for a second, which fact has enabled him to make many additions to the text as well as to the illustrations. He makes graceful acknowledgement to J. B. Murphy, W. J. Mayo and Mayo Robson for the benefit derived from their surgical work, to which he frequently alludes in the text.

In January, 1906, a notice of Moynihan's first edition appeared in these columns from which we extract the following paragraph:

The author presents his own experience in lucid fashion and quotes cases in support of his opinions, not only from his own practice but also from that of other surgeons of experience. The work is beautifully illustrated in large part from original drawings and is printed on heavy book paper, making it at once a handsome as well as a durable volume. It is a book which every abdominal surgeon will need, and its guidance will be found of value in many difficult conditions.

This statement is reaffirmed in regard to Mr. Moynihan's second edition. His earlier work is confirmed by his later experiences, all of which receives the stamp of approval at the hands of the surgeons on both sides of the Atlantic.

